# Impact of clinical pharmacist interventions on inappropriate prophylactic acid suppressant use in hepatobiliary surgical patients undergoing elective operations

**DOI:** 10.1371/journal.pone.0186302

**Published:** 2017-10-18

**Authors:** Hongli Luo, Qingze Fan, Shunlin Xiao, Kun Chen

**Affiliations:** Department of Pharmacy, the Affiliated Hospital of Southwest Medical University, Luzhou, China; University Hospital Llandough, UNITED KINGDOM

## Abstract

**Objective:**

To evaluate the impact and cost-benefit of clinical pharmacist interventions on inappropriate use of prophylactic acid suppressant in hepatobiliary surgical patients in a Chinese tertiary hospital.

**Methods:**

A retro-prospective intervention study of patients undergoing elective operations was performed in the Department of Hepatobiliary Surgery of the Affiliated Hospital of Southwest Medical University. Patients admitted from October to December 2015 and from October to December 2016, served as the pre-intervention and the post-intervention group, respectively. Clinical pharmacist interventions in the post-intervention group included real-time monitoring medical records and recommending that surgeons prescribe prophylactic acid suppressants according to the criteria established by the hospital administration. Then, the clinical outcomes of post-intervention group were compared with the pre-intervention group which lacked pharmacist interventions. In addition, cost-benefit analysis was conducted to determine the economic effects of implementing the clinical pharmacist interventions in acid suppressant prophylaxis in perioperative period.

**Results:**

Clinical pharmacist interventions significantly decreased the rate of the use of no indications for prophylactic acid suppressant and of the cases of inappropriate drug selection, dose, route, replacement and prolonged duration of prophylaxis (*P* < 0.05 or *P* < 0.001), resulting in significant increase by 10.65% in the percentage of cases adhering to all the criteria (*P* < 0.001). Moreover, significant reductions were found in the average usage quantity (*P*<0.001), mean cost (*P* = 0.03) and mean duration (*P* < 0.001) of prophylaxis acid suppressant. The ratio of the mean cost savings for acid suppressants to the mean cost of pharmacist time was 13.61:1.

**Conclusion:**

The clinical pharmacist’s real-time interventions facilitated the rational use of prophylactic acid suppressant and resulted in favorable economic outcomes in hepatobiliary surgery.

## Introduction

Stress ulcer or stress related mucosal disease that appears after major stressful events such as surgery, trauma and mental illness is superficial lesions commonly involving the mucosal layer of the stomach. Previous studies showed that a serious complication, stress ulcer bleeding is rare, but the risk is higher in intensive care unit (ICU) patients than non-ICU patients [1, 2]. Without stress ulcer prophylaxis (SUP), approximately 6% of critically ill patients experience clinically significant gastrointestinal bleeding (GIB) [3]. Another study showed no decrease in bleeding rate when using SUP for non-ICU patients [4]. Thus it has been validated that the SUP was beneficial for ICU patients, but this was not the case for non-ICU patients such as general surgery patients. However, overutilization of SUP in both ICU and non-ICU patients has become increasingly common recently [5, 6]. Overutilization is defined as prescribing SUP without a documented indication or inappropriate continuation upon discharge from the hospital. To assist clinicians with appropriate use of SUP, several organizations have developed clinical practice guidelines (CPGs) for SUP [7]. For example, SUP guidelines published in 1999 by the American Society of Health-System Pharmacists (ASHP) recommended that acid suppressants should only be used for patients with at least one present risk factor, such as coagulopathies, mechanical ventilation, history of gastrointestinal ulceration or bleeding, etc [8]. Furthermore, based on the national and local circumstance, the National Health and Family Planning Commission (NHFPC) of China and Health and Family Planning Commission of Sichuan Province have incorporated the guidelines into the national drug policy and local enforcement regulation.

Despite the availability of these CPGs and internal policies, the prophylactic use of acid suppressant is still far from optimization. A retrospective analysis found that 73% of patients were prescribed SUP without an appropriate indication, with 69% of patients continuing upon discharge [9]. A prospective study showed that 91.5% of patients in the infectious disease ward who received acid suppression therapy did not have an indication for SUP [10]. A considerable portion of surgeons did not adhere to the basic principles suggested by issued guidelines for SUP [11, 12]. Analogously, the inappropriate PPIs use in the perioperative period of surgical procedures was ubiquitous in the department of hepatobiliary surgery of the affiliated hospital of Southwest Medical University, located in Luzhou, China. Our previous study indicated that the rate of PPI prescribing was up to 84.04%, yet no indication usage was 77.77% in hepatobiliary surgery of our hospital [13].

Recommended agents for SUP were proton pump inhibitor (PPI) and histamine-2 receptor antagonist (H_2_RA). Overutilization of both H_2_RA and PPI poses significant health risks and increases healthcare costs. The uncontrolled and probably unnecessary utilization of PPI could lead to increased risk of avoidable adverse events (such as hospital /community—acquired pneumonia and *Clostridium difficile*-associated diarrhoea) and potential complications (acute interstitial nephritis, fracture risk, vitamin and mineral deficiency, hypomagnesemia, etc.) and drug interactions [14–17]. The clinic adverse events of H_2_RAs were mainly observed on the skin and the nervous system [18]. Therefore, to prevent these complications and avoid medical waste, it is greatly essential to implement interventions to improve the rational use of acid suppressant prophylactic.

Special pharmacists have become an established feature of the medical stewardship landscape in hospitals, particularly antibiotic specialist pharmacists [19, 20]. A multicentre, cluster randomized, controlled trial showed that special pharmacists could play important role in reducing the frequency of a series of clinically important prescription and medication monitoring errors [21]. Recently, studies showed that pharmacist intervention decreased PPIs use in non-ICU hospitalized patients and overutilization of SUP in medical and surgical ICU [22, 23]. A retrospective study discovered that the relative reduction in the rate of inappropriate SUP in general ward patients was 83.5% after implementing pharmacist SUP managed program [24]. However, in most Chinese hospitals, clinical pharmacists are not involved in controlling irrational use of prophylactic acid suppressant, so few data are available on the effectiveness of pharmacist interventions, especially studies about the cost-benefit results of introducing clinical pharmacist as a member of SUP team. So far, we have not found any published literatures focusing on the effects of clinical pharmacist interventions on the SUP prescribing in hepatobiliary surgery. In our retro-prospective study, to decrease the inappropriate use of prophylactic acid suppressant and to reduce acid suppressant costs, a clinical pharmacist was delegated to monitor the real-time use of prophylactic acid suppressant through an internal guideline for SUP in perioperative period. The purpose of this study was to evaluate the clinical and economic impacts of clinical pharmacist interventions for SUP at a Chinese tertiary teaching hospital.

## Methods

### Study design

A single-center retro-prospective study was performed on patients who underwent elective operations in the Department of Hepatobiliary Surgery of the Affiliated Hospital of Southwest Medical University, which is a 3000-bed major academic tertiary hospital with an average daily admission rate of about 3500 patients per day and > 120,000 inpatient admissions annually in Luzhou, China. This study included pre-intervention and post-intervention stages. Patients admitted during the 3-month period October–December 2015 and October–December 2016 undergoing elective operations in department of hepatobiliary surgery were assigned to the pre-intervention and the post-intervention group, respectively. The pre-intervention stage was an observational period for retrospective study, in which clinical pharmacist collected medical records of the patients and discovered problems associated with acid suppressant prophylaxis in perioperative period. In the post-intervention stage, a full-time, experienced clinical pharmacist worked in the ward. The rationality of SUP in these patients was evaluated before and after the implementation of clinical pharmacist intervention program. This program was approved by the Hospital Pharmacy Administration and Therapeutics Committee (HPATC), which granted clinical pharmacist to make suggestions to the doctors, but not to modify doctor's advice when inappropriate prescriptions occurred.

All adult (≥18 years) patients undergoing elective operations in the hepatobiliary ward were enrolled. Patients were included if they had undergone hepatobiliary surgery and had no systemic diseases. Patients were excluded if they had undergone emergency operation or any invasive operation within one month prior to hepatobiliary surgery. Also, patients were excluded if acid suppressant was prescribed for treatment of gastrointestinal diseases (eg, gastrointestinal reflux disease, peptic ulcer disease, Zollinger-Ellison or gastrointestinal hemorrhage) or taking acid suppressant within two weeks prior to surgery regardless of whether an indication was documented in the medical chart. Patients transferring from other medical departments or transferring to other medical departments for further treatment were excluded from analysis. In the pre-intervention stage, all medical records from October to December 2015 were exported and we randomly took samples of 250. Randomization was computer-generated. In the post-intervention stage, according to established inclusion criteria, we recruited this number (250) then stop. All eligible patients in the post-intervention stage received the clinical pharmacist intervention from October to December, 2016.

With reference to ASHP protocol, China Consensus guidelines and official document for SUP, an internal guideline for SUP in perioperative period was prepared by clinical pharmacists. After HPATC approval, the clinical pharmacist checked the prescription of every patient and advised the surgeons in charge on prescribing acid suppressant for SUP in situations when there was an indication or advised the discontinuation of acid suppressant when not warranted by patient risk factors. The indicators for rational use of prophylactic acid suppressant were judged based on the criteria as shown in Table 1.

**Table 1:** Internal guideline for SUP in perioperative period in patients on the hepatobiliary surgery ward of the Affiliated Hospital of Southwest Medical University.

Parameter	Justification for rational use
Indications	One of the following risk factors:
	Severe traumatic brain or spinal cord injury (Glasgow Coma score of ≤ 10)
	Thermal injury to > 35% of body surface area
	Severe multiple trauma (injury severity score of ≥ 16)
	Difficult or complex surgery (operative time > 3 hours)
	Severe psychological stress
	Hepatic or renal failure
	Mechanical ventilation for > 48 hours
	Coagulopathy (platelet count of < 50 000 mm^3^ or INR > 1.5 or APTT > 2 times normal value)
	History of gastrointestinal ulceration or bleeding within 1 year of admission
	Dual antiplatelet therapy
	Two or more of the following risk factors: ICU stay of greater than 1 week, occult bleeding lasting at least 6 days, high-dose use of corticosteroids (> 250 mg/d of hydrocortisone or equivalent daily), or use of non-steroidal anti-inflammatory drugs (NSAIDs)
Agents	H_2_RA (low-risk for GIB), PPI (high-risk for GIB)
Dosage	Dose must be based on risk factors and concentration used for surgical prophylaxis purposes for each acid suppressant
Route	Route must be based on risk factors and surgical patients’ condition
Duration of prophylaxis	Until no high risk factors, or able to tolerate enteral feeding, or not receiving mechanical ventilation or not in ICU

### Pharmacist interventions

**Fig 1:**
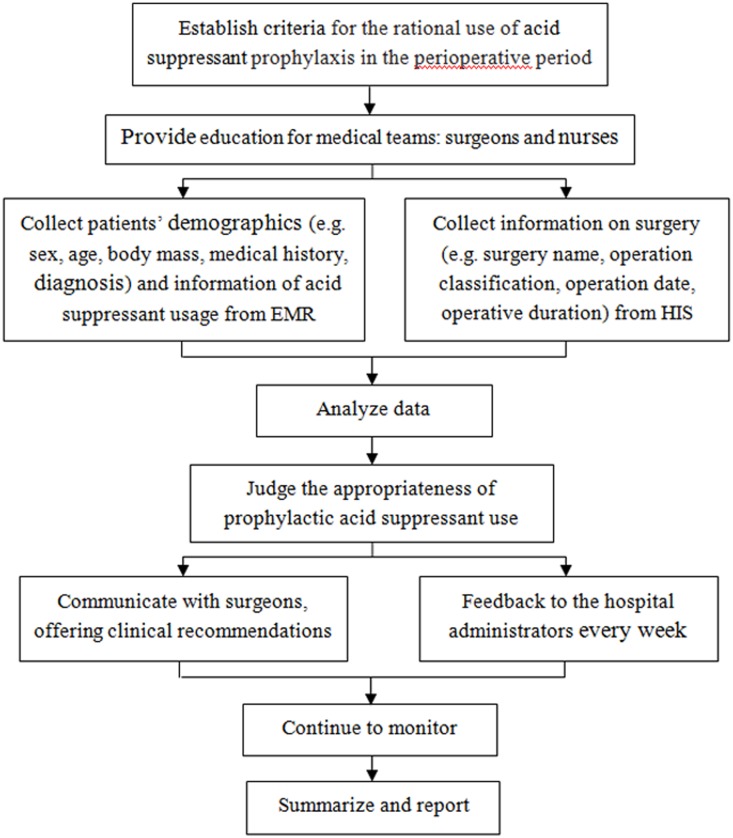
Flow chart of clinical pharmacist intervention. HIS, hospital information system; EMR, electronic medical records.

The pharmacist intervention was implemented by our previous method with some modifications [25]. During the intervention period, there was an appropriative clinical pharmacist in the hepatobiliary surgery ward. On average, the clinical pharmacist spent 4 hours on the interventions every work day. The interventions were endorsed by HPATC and the leadership of hepatobiliary surgery department, and implemented from October to December 2016. The job responsibilities of the clinical pharmacist included correcting the surgeons’ misunderstanding, monitoring medical records in real time and controlling the prescriptions of prophylactic acid suppressant on the basis of the criteria established by the hospital administration. The clinical pharmacist interventions consisted of: (1) Providing educational sessions and handouts about SUP for medical teams, especially the surgical residents who prescribed acid suppressant and the nurses who executed prescriptions, including knowledge about acid suppressants’ indications, pharmacokinetics, pharmacodynamics, strength and duration. The appropriate administration route and dose were executed by nurses; (2) Before the elective operation and after the surgeons prescribed the medication, clinical pharmacist collected the information about surgical patient from electronic medical records (EMR) and surgery from hospital information system (HIS), and judged the appropriateness of the use of prophylactic acid suppressant on following aspects: indication, acid suppressant selection, dose, duration of prophylaxis, combination and replacement depending on our criteria. When obviously irrational use of prophylactic acid suppressant was identified, the clinical pharmacist communicated immediately with surgeons and provided recommendations to correct the medication errors. (3) The inappropriate orders of SUP were collected and categorized by the clinical pharmacist every day and the identified problems were reported to the hospital administration every week. The workflow of clinical pharmacist was illustrated in Fig 1.

### Data collection and analysis

The data were collected from medical records of patients, containing patients’ demographics (sex, age, body mass, medical history, diagnosis and allergies), surgical procedures (name, date and duration of operation), acid suppressant usage (generic name, dose, route, frequency, duration, combinations and replacement) and cost (costs of total hospitalization, total drugs and acid suppressant). The data collection was conducted by another clinical pharmacist who was blinded to the patients’ allocation status. All costs were recorded in Chinese yuan and then converted to US dollars (exchange rate, 6.96 yuan = US $1). The final values were reported in US dollars.

Cost-benefit analysis was performed to determine the economic effects of implementing clinical pharmacist interventions for perioperative acid suppressant prophylaxis. The analysis was an evaluative technique of comparing the costs of resources consumed in implementing clinical pharmacist interventions against the benefits resulted from the interventions. The benefit-to-cost ratio was calculated by dividing the acid suppressant cost saving by the cost of time spent by clinical pharmacist. The mean acid suppressant cost saving and the mean cost of pharmacist time were applied because the numbers of patients in two groups were not equal [19]. The mean acid suppressant cost saving was gained by calculating the difference in mean cost of acid suppressant between the pre- and post-intervention phases. The mean cost of pharmacist time was calculated according to the hourly salary of clinical pharmacist and the amount of time spent by clinical pharmacist in implementing the interventions.

Data were entered and subsequently analyzed using SPSS version 22.0. For comparison between the two stages, data were analyzed using chi-squared test for categorical variables, and using student’s t-test for continuous variables to assess the significant statistical differences. A p-value of less than 0.05 was considered statistically significant.

### Ethics statements

This retro-prospective study was approved by the Ethics Committee of the Affiliated Hospital of Southwest Medical University. All patients provided written informed consent to permit their information to be stored in the hospital database and used for this study.

## Results

### General characteristics of the patients

**Fig 2:**
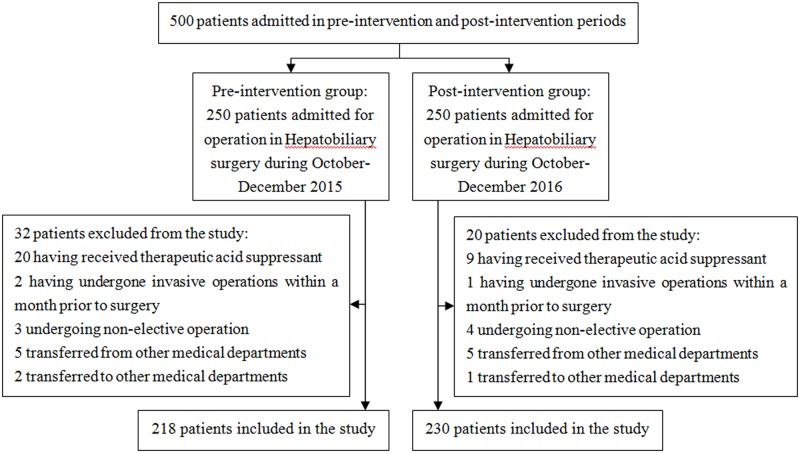
Patients selection flow chart.

In the beginning, A total of 500 patients were enrolled in this study, 250 patients in each group, Then, 52 patients who didn’t meet our criteria were excluded as shown in Fig 2. At last, 448 patients in total were included for further research with 218 in the pre-intervention group and 230 in the post-intervention group as shown in Fig 2. General characteristics of the patients in two groups were shown in Table 2. The 2 groups were similar with respect to demographics and clinical characteristics, such as sex, age, body mass, medical history, surgery type and operative duration (*P* > 0.05). There was no significant difference in post-operative bleeding between the two groups (*P* > 0.05) (Table 2). Three cases received therapeutic acid suppressant after surgery, so they were excluded when analyzing the rationality of prescriptions and the cost-benefit results.

**Table 2:** General characteristics of patients in pre- and post-intervention groups.

Characteristics	Pre-intervention (n = 218)	Post-intervention (n = 230)	*p*-value
Male, n (%)	78 (35.78)	78 (33.91)	NS^a^
Age, mean ± SD	57.70±13.60	53.16±14.07	NS
Old (>65 years), n (%)	69(31.65)	55(23.91)	NS
Body mass (kg)	62.15±10.04	65.37±12.81	NS
Type of surgery, n (%)			
Laparoscopic cholecystectomy (LC)	112 (51.38)	105 (45.65)	NS
Open cholecystectomy (OC)	46 (21.10)	37 (16.09)	NS
Bile duct exploration	5 (2.29)	13 (5.65)	NS
Hepatectomy	21 (9.63)	27 (11.74)	NS
Liver cyst fenestration	9 (4.13)	18 (7.83)	NS
Resection of the hepatic hemangioma	9 (4.13)	14 (6.09)	NS
Splenectomy	9 (4.13)	5 (2.17)	NS
Pancreaticoduodenectomy	3 (1.38)	9 (3.91)	NS
Operative time, n (%)			
>4 hours	25 (11.47)	32(13.91)	NS
Post operative GI bleeding, n (%)	2 (0.92)	1 (0.43)	NS

^a^NS = not significant (*P* > 0.05)

### Indications and rate of prophylactic usage

According to the established criteria for SUP in perioperative period, 38 cases and 48 cases showed indications for SUP in the pre- and post-intervention groups, respectively. However, 216 cases (100%) and 169 cases (73.80%) received SUP in pre- and post-intervention groups, respectively. In the post-intervention group, 46 cases with indications for SUP were included among the 169 cases that actually received SUP. There was a significant decrease in the rate of acid suppressant prophylaxis in the post-intervention group (Table 3).

**Table 3:** Indications for SUP and receipt of acid suppressant prophylaxis.

Prophylaxis with H_2_RA or PPI	Indication for SUP					
	Pre-intervention			Post-intervention		
	Yes	No	Total	Yes	No	Total
Yes	38 (100%)	178 (100%)	216 (100%)	46 (95.83%)	123 (67.96%)	169 (73.80%)
No	0	0	0	2 (4.17%)	58 (32.04%)	60 (26.20%)
Total	38 (100%)	178 (100%)	216	48 (100%)	181 (100%)	229

### Frequency of prophylactic acid suppressant usage

There were combinations or replacements of acid suppressant in some cases, which caused the same patient to be administered greater than or equal to 2 types of acid suppressant and the frequency of acid suppressant utilization to be higher than the number of patients. The frequency of prophylactic acid suppressant usage was 285 in the pre-intervention group and 186 in the post-intervention group. Significant reduction in mean number of acid suppressant used was observed after the intervention (*P* < 0.001). As shown in Table 4, the prescription rates of H_2_RAs and PPIs were 0% and 13.44%, 100% and 86.56% in pre- and post-intervention group, respectively. A high rate of omeprazole prescription was observed in two stages. In the pre-intervention group, omeprazole and lansoprazole accounted for 95.44% of the acid suppressants used, followed by esomeprazole (4.56%). In the post-intervention group, omeprazole and lansoprazole accounted for 78.49% of the acid suppressants used, followed by famotidine (13.44%), pantoprazole (5.91%), esomeprazole (2.16%) (Table 4).

**Table 4:** Prophylactic acid suppressant use in pre- and post-intervention groups.

	Pre-intervention (n = 216)	Post-intervention (n = 229)	*p*-value
Frequencies of prophylactic acid suppressant usage	285	186	-
Histamine 2 receptor antagonists, n (%)	0	25 (13.44)	<0.001
famotidine	0	25 (13.44)	-
Proton pump inhibitors (PPIs), n (%)	285 (100)	161 (86.56)	<0.001
omeprazole	151 (52.98)	105 (56.45)	-
lansoprazole	121 (42.46)	41 (22.04)	-
pantoprazole	0	11 (5.91)	-
esomeprazole	13 (4.56)	4 (2.16)	-
Mean number of acid suppressant used	1.32	1.10	<0.001

### Rate of inappropriate prophylactic acid suppressant use

According to the established criteria, inappropriate prophylactic acid suppressant uses in the pre- and post-intervention groups were summarized in Table 5. After intervention, obvious improvements were observed in the use of no indications for prophylactic acid suppressant and in the cases of inappropriate drug selection, dose, route, replacement and prolonged duration of prophylaxis (P < 0.05), but the proportion was not satisfied in both groups. When coadministration of omeprazole or esomeprazole with clopidogrel, generation of clopidogrel active metabolite and inhibition of platelet function were reduced, so omeprazole or esomeprazole should try to avoid using when combining with clopidogrel [26]. Five cases in pre-intervention group were inappropriately prescribed the combination of omeprazole plus clopidogrel, but this combination was not found in post-intervention group.

**Table 5:** Rate of inappropriate prophylactic acid suppressant use in surgical patients for SUP.

	Pre-intervention n (%)	Post-intervention n (%)	*p*-value
No indication	178 (82.41%)	123 (72.78%)	0.023
Inappropriate choice of acid suppressant	5 (2.32)	0 (0.00)	0.046
Inappropriate dose	31 (14.35)	12 (7.10)	0.025
Inappropriate administration route	132(61.11)	63 (37.27)	<0.001
Repeated medication	3 (1.39)	0 (0.00)	NS^a^
Unnecessary replacement of drugs	56 (25.93)	17(10.06)	<0.001
Unnecessary prolonged duration of prophylaxis	141 (65.28)	64 (37.87)	<0.001

^a^NS = not significant (*P* > 0.05). No indication refers to cases administrated PPIs without risk factors as shown in Table 1. Inappropriate administration choice of acid suppressant refers to the coadministration of omeprazole or esomeprazole with clopidogrel. The inappropriate administration route involves unnecessary intravenous administration when oral formulations would be more appropriate and confusing misuse of intravenous drip and intravenous injection. Repeated medication refers to cases in which patients take more than 2 PPI prescriptions at the same time. Unnecessary replacement of drugs refers to the conditions where the acid suppressant selection was discordant between the first dose and postoperative maintenance.

### Rate of correct acid suppressant administration

As shown in Table 6, a total of 17.59% of cases in the pre-intervention group and 27.22% of cases in the post-intervention group complied with the established internal criteria for the indication. None of 216 cases adhered completely to all the five criteria (indication, choice, dose, route and duration) in pre-intervention group. However, pharmacist interventions led to a remarkable increase by 10.65% in the percentage of cases adhering to all the criteria (P < 0.001). But the percentage of compliance to all the five criteria in post-intervention group was not satisfied, which was due to a high rate of incorrect administration route and prolonged duration of acid suppressant prophylaxis (Table 6).

**Table 6:** Rate of correct* acid suppressant administration in surgical patients for SUP.

Acid suppressant administration	Pre-intervention n (%)	Post-intervention n (%)	*p*-value
Correct indication	38 (17.59)	46 (27.22)	0.023
Correct indication+ correct choice	35 (16.20)	46 (27.22)	0.008
Correct indication+ correct choice+ correct dose	26 (12.04)	44 (26.04)	<0.001
Correct indication+ correct choice+ correct dose+ correct route	15 (6.94)	31 (18.34)	<0.001
Correct indication+ correct choice+ correct dose+ correct route+ correct duration	0 (0.00)	18 (10.65)	<0.001

correct* = ‘correct’ was defined as the indication, choice, dose, route or duration which met the criteria of internal guideline for SUP (Table 1).

### Cost-benefit analysis of clinical pharmacist intervention

There was no change in the price of acid suppressants, other drugs and hospital service during the study period. There were no significant differences between the two groups regarding mean total hospitalization cost, mean total drug cost and mean hospitalization days (P > 0.05). Significant reductions in mean acid suppressant cost (P < 0.05) and mean duration of prophylaxis acid suppressant (P < 0.001) were observed in post-intervention groups (Table 7). The mean acid suppressant cost savings were $83.32 in the intervention stage. The total work time of the clinical pharmacist was 264 hours during 3-month intervention period. With the hourly salary of $5.31, the total cost of the pharmacists’ time was approximately $1401.84 during intervention period. The mean cost of the pharmacists’ time was calculated as the ratio of the total cost of the pharmacist’s time to the total number of cases, accordingly, yielding a mean value of $6.12. Therefore, the intervention resulted in a considerable benefit-to-cost ratio of 13.61:1 (Table 8).

**Table 7:** The medical cost in pre- and post-intervention groups.

	Pre-intervention (n = 216)	Post-intervention (n = 229)	*p*-value
Mean total hospitalization cost (USD)	4359.71	4106.52	NS^a^
Mean total drug cost (USD)	1111.57	1055.44	NS
Mean acid suppressant cost (USD)	161.59	78.27	0.030
Mean duration of prophylaxis acid suppressant (day)	9.48	4.68	<0.001
Mean hospitalization days	13.45	12.50	NS

^a^NS = not significant (*P* > 0.05)

**Table 8:** Cost-benefit analysis of clinical pharmacist intervention.

Cost of clinical pharmacist time	
Hourly salary	$5.31
4 hours per working day × 66 working days during intervention period	264 hours
Total cost of pharmacist time (264 hours × $5.31 per hour)	$1,401.84
Mean cost of pharmacist time (total cost of pharmacist time ÷229 cases)	$6.12
Mean acid suppressant cost reduction for 229 cases in the post-intervention group	
Mean acid suppressant cost for 216 cases in pre-intervention group—Mean acid suppressant cost for 229 cases in the post-intervention group	$83.32
Net cost benefit	
Mean acid suppressant cost reduction—Mean cost of pharmacist time	$77.20
Benefit-to-cost ratio	
Mean acid suppressant cost reduction: mean cost of pharmacist time	13.61:1

## Discussion

Despite growing concerns about the utilization of SUP in surgical patients, we believe that this is the first published report demonstrating improved rational use of acid suppressant prophylaxis and increased cost savings after implementation of clinical pharmacist intervention in hepatobiliary surgery.

In the present study, no indication use of acid suppressant was reduced after intervention, which demonstrated the important role of pharmacists in making prescriptions of SUP accord with the indications. However, only 27.22% of patients had an indication for SUP but 73.80% received acid suppressants in the post-intervention stage, which was similar to other recently published studies [27, 28]. At the same time, no indication use for SUP was common in many hospitals in Lebanese, USA, Switzerland, Iran [10, 12, 29, 30]. Recently published studies found that the frequency of clinically important bleeding reported was low and there was little significant reduction in bleeding with medication prophylaxis. Moreover, the side effects of PPI and H_2_RA are not insignificant, and it is not cost-effective to use acid-suppressive therapy to prevent GIB except in the high-risk patients [23, 31]. It was confirmed that acid suppressants for SUP did not significantly reduce GIB in our study. Therefore, the pharmacist intervention could not only reduce the waste of medical resources on account of the fact that most of the prescriptions were useless, but also avoid the side effect resulting from the unnecessary use of SUP.

We proposed that there are two major factors that cause the overuse of acid suppressant in the hospital in China. On the one hand, there remained some misunderstandings about SUP in the surgeons. Before the intervention program was implemented, we conducted a survey of the surgeons about the characteristics and side effects of acid suppressant and found some misconceptions such as “acid suppressants for SUP resulted in a lower rate of post-surgical GIB” and “when PPIs and H_2_RAs were applied for a short term, no side effects would be observed”. On the other hand, the tense doctor-patient relationship remained unchanged for decades in China. To protect themselves from lawsuits, surgeons had to prescribe additional acid suppressant to minimize the possibility of GIB. In view of this fact, the number of clinical pharmacists and working hours should be increased, which would contribute to more extensive medication education and in-depth communication with surgeons and patients.

Recommendations on agents for SUP were not consistent across developed high quality CPGs. The most of CPGs recommended using both PPI and H_2_RA. The previous studies reported that PPI was the most frequently used acid suppressant for SUP [32, 33]. While, another study found that providing SUP with H_2_RA therapy may reduce costs, increase survival, and avoid complications compared with PPI therapy [34]. Our guideline also recommended H_2_RA as the preferred agent over PPI for low-risk patients for GIB in perioperative period, especially when combining with clopidogrel [35]. In our results, pharmacist intervention could avoid the inappropriate choice of omeprazole and esomeprazole and increase the usage of safer and cheaper H_2_RA (Table 4). Nevertheless, the usage rate of H_2_RA only accounted for 13.44% in post-intervention period because of surgeons’ misconceptions, such as “new or expensive drugs are stronger”. So, more efforts should be done in the future.

The inappropriate route and duration was more serious in our study (Table 5). The improper administration route or prolonged acid suppressant prophylaxis is, at best, of no benefit and, at worst, unnecessary drug cost and potentially harmful to patients because of the toxicity and the risk of complications. Possible reason for the high ratio of inappropriate route in our study may be that our hospital has two different brands of omeprazole for different injection method, which is easy for confusion. The one produced by AstraZeneca Pharmaceutical should be used by intravenous injection, while, the other produced by Jiangsu Aosaikang Pharmaceutical company should be used via intravenous drip. In order to solve this problem, some methods could be adopted in the future such as pasting another eye-catching label when these drugs are distributed or changing the medicines to another brands to unify the administration route. On the other hand, several CPGs recommended that SUP should be discontinued when there was no risk factor for stress ulcer or the patient could tolerate enteral feeding. Because of the negligence of the duration under the heavy operation work and the misconception of that “the longer duration, the better efficacy”, surgeons always prolonged the duration of prophylaxis. Hence, pharmacist should pay more attentions on the duration and remind the surgeons when the drugs should be discontinued as soon as possible.

Based on our analysis, the clinical pharmacist intervention could save the unnecessary SUP costs and the benefit-to-cost ratio was almost 13:1, showing an economically beneficial effect for both the hospital and patients. In the past, the “drug-maintaining-medicine” has long been a particular character in the health care system in China, which means covering hospital expenses with medicine revenue. However, this old system caused some serious problems including the waste of medical resources, the unreasonably excessive profit of pharmaceutical factory, the serious economic burden of patients and more seriously, the side effects and harm to patients’ health. In order to solve these problems, many policies are implemented in China to change the hospital’s reliance on drugs sales such as the zero bonus of drugs. In this kind of environment, the cost benefit of clinical pharmacists could play a very important role for saving costs of medicines. Moreover, in developing countries like China, the ratio of patients to doctors is very high, which means that the doctors have very heavy work for diagnose and operation. Thus, it’s very difficult for doctors to focus on the rational use of drugs, especially for surgeons. Therefore, pharmacist intervention could help check the rationality of prescription and release these burdens of doctors, guaranteeing the efficacy of drugs, safety of health and economical efficiency of patients.

Even if many advantages of pharmacist intervention in China, clinical pharmacists are only able to make suggestions to the doctors or refuse to dispense prescriptions when inappropriate prescriptions are identified. If the doctors refuse to accept their suggestions, their intervention could not play its role. During the first month of the intervention stage, the rate of surgeons with resistance to the pharmacist’s advices reached approximately 70% because of the surgeons’ misconceptions or prescribing habits. With the efforts of pharmacist’s continual communications, most of the surgeons gradually recognized the disadvantages of inappropriate use of prophylactic acid suppressant and finally accepted the pharmacist’s suggestions, which led to the reduction of resistance rate to approximately 30% during the later stages. Even though, the surgeons were not willing to completely accept the clinical pharmacist’s advice. Thus, it was difficult for the clinical pharmacist to further reduce the irrational use of prophylactic acid suppressant. In order to maximize the effect of clinical pharmacist intervention, hospital administrators should increase the number of clinical pharmacists or prolong the working time of clinical pharmacists to enhance communication between clinical pharmacists and surgeons, and to extend this intervention to other surgical departments. Moreover, clinical pharmacists should be authorized to deny irrational prescriptions.

Unfortunately, our study had several limitations. First, this intervention study was performed on the basis of a pre-to-post design without involving a simultaneous control group, so this retro-prospective study was less convincing than a prospective, controlled study design. Second, this was a single center and a single post-implementation period evaluation of SUP managed by clinical pharmacists. Ideally, another post-implementation period (ie, fourth quarter 2017) may be needed to confirm a sustained impact. Third, although we observed a significant reduction of inappropriate SUP in hospitalized patients in hepatobiliary surgery during intervention phase, the result (10.65%) was not satisfied. We will take further measures to rectify administration route and shorten SUP duration in the future. Lastly, we utilized identical time periods to eliminate any potential seasonal influence, but we did not identify any other factors that could potentially have influenced the use of acid suppression agents such as shortage of medicine and turnover of surgeons in this teaching hospital during the study periods. These favorable results obtained could not be attributed solely to the clinical pharmacist intervention. Therefore, the reliability of clinical pharmacist intervention needs to be confirmed in more rigorous studies.

## Conclusion

The present study demonstrated that implementation of real-time clinical pharmacists intervention for SUP in hepatobiliary surgery improved the appropriate utilization of acid suppressants as well as resulted in favorable economic outcomes. Considering the cost-benefit value, hospital administrators should prolong working time of clinical pharmacists or increase the number of clinical pharmacists to monitor prophylactic acid suppressants. In the future, clinical pharmacists would try their best to expand pharmacists’ practice scope to improve prescription rationality and medication monitoring in general practices.
